# Detection error influences both temporal seroprevalence predictions and risk factors associations in wildlife disease models

**DOI:** 10.1002/ece3.5558

**Published:** 2019-08-27

**Authors:** Michael A. Tabak, Kerri Pedersen, Ryan S. Miller

**Affiliations:** ^1^ Center for Epidemiology and Animal Health United States Department of Agriculture Fort Collins Colorado; ^2^ Wildlife Services United States Department of Agriculture Raleigh North Carolina

**Keywords:** detection probability, hierarchical Bayesian model, opportunistic sampling, pathogen, prevalence, sensitivity, specificity, wildlife

## Abstract

1. Understanding the prevalence of pathogens in invasive species is essential to guide efforts to prevent transmission to agricultural animals, wildlife, and humans. Pathogen prevalence can be difficult to estimate for wild species due to imperfect sampling and testing (pathogens may not be detected in infected individuals and erroneously detected in individuals that are not infected). The invasive wild pig (*Sus scrofa*, also referred to as wild boar and feral swine) is one of the most widespread hosts of domestic animal and human pathogens in North America.
2. We developed hierarchical Bayesian models that account for imperfect detection to estimate the seroprevalence of five pathogens (porcine reproductive and respiratory syndrome virus, pseudorabies virus, Influenza A virus in swine, Hepatitis E virus, and *Brucella* spp.) in wild pigs in the United States using a dataset of over 50,000 samples across nine years. To assess the effect of incorporating detection error in models, we also evaluated models that ignored detection error. Both sets of models included effects of demographic parameters on seroprevalence. We compared our predictions of seroprevalence to 40 published studies, only one of which accounted for imperfect detection.
3. We found a range of seroprevalence among the pathogens with a high seroprevalence of pseudorabies virus, indicating significant risk to livestock and wildlife. Demographics had mostly weak effects, indicating that other variables may have greater effects in predicting seroprevalence.
4. Models that ignored detection error led to different predictions of seroprevalence as well as different inferences on the effects of demographic parameters.
5. Our results highlight the importance of incorporating detection error in models of seroprevalence and demonstrate that ignoring such error may lead to erroneous conclusions about the risk associated with pathogen transmission. When using opportunistic sampling data to model seroprevalence and evaluate risk factors, detection error should be included.

## Graphical abstract

We estimated the prevalence of five pathogens, and their associated risk factors, in a widespread invasive species using Bayesian models that account for imperfect detection. We compared models that did and did not account for detection error and found that they resulted in different inferences about prevalence and risk factors associated with prevalence. This paper highlights the importance of accounting for detection error in disease models.

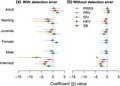

## 1 INTRODUCTION

Pathogens transmitted among humans, wildlife, and domestic animals have increasingly received attention because of the emergence of pathogens causing disease in humans, economic damage to agricultural systems, and conservation concerns for wildlife (Miller, Farnsworth, & Malmberg, 2013; Miller et al., 2017; Wiethoelter, Beltrán‐Alcrudo, Kock, & Mor, 2015). Disease transmission at the human–domestic animal–wildlife interface is inherently complex, and mitigating transmission risks requires understanding the role of wildlife in the epidemiology, spread, and maintenance of infectious diseases (Hassell, Begon, Ward, & Fèvre, 2017). Central to these goals, and often required for successful management or eradication of these diseases, are accurate predictions of disease prevalence (Pepin et al., 2017). Previous work has shown that information on detection error (e.g., diagnostic assay sensitivity and specificity) can be incorporated into models using serosurveillance data, reducing bias in predictions of seroprevalence and risk factors (DiRenzo et al., 2018; McClintock et al., 2010; Miller, Talley, Lips, & Grant, 2012). Yet, there remains a limited number of studies that explicitly account for detection error when predicting disease prevalence and the potential effect on inference of risk factors is not available.

Analysis of the temporal occurrence and distribution of disease plays an important role in epidemiology (Vergne, Gogin, & Pfeiffer, 2017). Prevalence estimates for wildlife populations are often based on opportunistic samples from animals due to the difficulties in capturing and collecting samples. Apparent prevalence, the number of animals that test positive divided by the total number tested, is often not a useful estimate of the underlying disease prevalence due to unbalanced sample sizes, differences in diagnostic assay use, and variation across time (Pepin et al., 2017). The elucidation of temporal patterns is often complicated by missing or incomplete data, which is a common occurrence for wildlife populations (Kodric‐Brown & Brown, 1993). However, in the field of wildlife disease, the uncertainty associated with the diagnostic testing process is rarely included in published predictions of prevalence in wildlife. This is an important issue, as most diagnostic assays are developed for domestic animals and are not validated for wildlife (Stallknecht, 2007). Assays that have been evaluated for wildlife often have significantly different diagnostic uncertainty (Gardner, Hietala, & Boyce, 1996). This uncertainty may affect estimates of disease or pathogen prevalence and, in turn, estimates of risk factors associated with the host–pathogen system.

A primary focus of disease ecology has been to identify correlations between the temporal distribution of disease and demographic variables for natural populations (Delahay, Langton, Smith, Clifton‐Hadley, & Cheeseman, 2000; Farnsworth et al., 2012; Osnas, Heisey, Rolley, & Samuel, 2009). One application of these correlations is identifying risk factors such as age or sex that are associated with higher rates of disease. Risk factors are frequently used to prioritize surveillance in wildlife when monitoring for pathogens of consequence to humans, domestic animals, or of conservation concern for wildlife (Heisey, Jennelle, Russell, & Walsh, 2014; Jennelle et al., 2018). Despite a robust literature examining risk factors associated with disease in wildlife, few have included true and false detection probabilities in models of pathogen prevalence. Recent studies have proposed the need to include true and false detection probabilities (i.e., the probability that a disease is detected when it is present and the probability that a disease is not detected when it is absent) in ecological models of disease (Lachish, Gopalaswamy, Knowles, & Sheldon, 2012; McClintock et al., 2010; Royle & Link, 2006). Several studies have demonstrated that not accounting for imperfect detection can result in underestimates of pathogen prevalence (DiRenzo et al., 2018; Lachish et al., 2012; Miller et al., 2012). Additionally, two of these studies demonstrated that imperfect detection is related to host infection intensity resulting in nonrandom bias in pathogen detection (DiRenzo et al., 2018; Lachish et al., 2012). Despite recently developed methods to account for imperfect detection in models of pathogen or disease prevalence and demonstrated effects on prevalence estimates, no study to date has evaluated the effect of imperfect detection on interpretation of risk factors. This can be particularly important for designing national‐scale monitoring and surveillance programs using risk factors to target surveillance intended to mitigate risks posed by wildlife disease (Gardner et al., 1996).

Here, we use pathogen serosurveillance data for invasive wild pigs in the United States to test hypotheses about the effect of demographic variables on the probability of infection for five pathogens of importance to human, domestic animal, and wildlife health (porcine reproductive and respiratory syndrome virus [PRRS], pseudorabies virus [PRV], Influenza A virus in swine [IAVS], hepatitis E virus [HEV], and *Brucella* spp. [SB]). Wild pigs in North America are considered one of the most important host species for transmission of pathogens to humans, domestic animals, and wildlife (Bevins, Pedersen, Lutman, Gidlewski, & Deliberto, 2014; Miller et al., 2017) receiving significant policy implementation to mitigate disease risks (Miller, Opp, & Webb, 2018). Since previous studies have found demographic factors (age and sex) to be associated with increased or decreased probability of infection (e.g., Cleveland et al., 2017; Feng et al., 2014), we evaluated these effects on seroprevalence. Additionally, we assessed the effect of accounting for detection error in models of seroprevalence and how this may affect interpretation of risk factor associations. We then conducted an extensive literature search to determine whether the patterns we found were consistent with previously published studies that did not account for imperfect detection. Our goals were to evaluate the significance of demographic factors, determine the effect of detection error on interpretation of these demographic factors, and to provide national‐scale estimates of the temporal true pathogen seroprevalence for the five pathogens investigated. Our results have broad implications for determining risk factor associations that can be used to inform disease management and risk‐based targeting in national‐scale surveillance programs.

## 2 MATERIALS AND METHODS

### 2.1 Surveillance data

We selected five pathogens that are important to human, domestic animal, and wildlife health. PRV causes spontaneous abortions, juvenile mortality, and respiratory illness in domestic pigs (Lari et al., 2006). PRV also causes rapidly fatal infections in livestock (Müller et al., 2011) and carnivores and is a threat to the endangered Florida panther (*Puma concolor coryi*; Glass et al., 1994). SB is an economically important disease of domestic pigs that can also be transmitted to humans and cattle (Olsen & Tatum, 2017). HEV has recently emerged as an important human health threat, as it is transmitted among humans and swine (Salines, Andraud, & Rose, 2017). PRRS is an important cause of late‐term reproductive losses, severe pneumonia, and increased mortality of domestic pigs with an estimated annual loss of $664 million to the US domestic swine industry (Holtkamp et al., 2013). IAVS can cause sporadic infections and pandemic outbreaks among humans and reduces the production of domestic swine (Ma, Kahn, & Richt, 2009).

We used data collected as part of the United States Department of Agriculture, Wildlife Services' disease surveillance in invasive wild pigs that are culled during agency control operations for damage management purposes. Serum is collected from culled animals annually for serologic monitoring of diseases of importance for human and animal health. Sampling is distributed throughout the United States with samples being collected throughout the year (Brown et al., 2019). The serological data used in our study were collected from 33,794 wild pigs in 845 counties in the United States from January 2007 through July 2018 (Appendix S1). The data include serological assay results for the five pathogens along with sex and age of the animal. Age class was determined at the time of sampling based on lower jaw tooth eruption, a common approach for wild pigs (Matschke, 1967); individuals were categorized as juvenile (<2 months), subadult (>2 months and ≤1 year), and adult (≥1 year). Samples were submitted to one of eight accredited veterinary diagnostic laboratories in the United States for serological testing. The diagnostic tests used for each pathogen are described in Appendix S2.

### 2.2 Model of pathogen prevalence

Observations from diagnostic test results (i.e., 0 is negative and 1 is positive) for each individual in each year (*y_it_*) were defined as:

$$y_{\mathrm{it}}∼\text{Bernoulli}\left(z_{\mathrm{it}}\rho +\left(1-z_{\mathrm{it}}\right)\left(1-\phi \right)\right)$$

where *z_it_* is the unobserved, latent infection state of individual *i* in year *t. ρ* is the sensitivity or the probability of detecting the pathogen when present (Pr(*y_it_* = 1|*z_it_* = 1)). Specificity (*ϕ*) is the probability that the pathogen is not detected when absent (Pr(*y_it_* = 0|*z_it_* = 0)). Note that individuals were only sampled once, so for each *i*, there is only one *t*. We use the two subscripts to clarify that seroprevalence estimates were calculated by year. National‐level seroprevalence in each year (*π_t_*) was calculated as the median *z* across all individuals and across all iterations in that year: (*π_t_* = median(*z_it_*)).

The latent unobserved disease state, *z_it_*, is a function of the probability that an individual is seropositive for the pathogen (*ψ_it_*):

$$z_{\mathrm{it}}∼\text{Bernoulli}\left(\psi_{\mathrm{it}}\right)$$

and *ψ_it_* is a function of demographic parameters:

$$\text{logit}\left(\psi_{\mathrm{it}}\right)=x_{i}^{T}\beta$$

where ***β*** is a vector of regression coefficients corresponding to $x_{i}^{T}$, which is the transpose of the vector of the demographic covariates of the *i*th individual. Parameters used in the model were the age and sex of each individual. The full model included age (i.e., juvenile, subadult, or adult), sex (i.e., male or female), and an intercept term. We also ran three reduced models for each pathogen including only age, only sex, and only intercept.

### 2.3 Prior distributions

Each of the regression coefficients was modeled using a vague prior:

$$\beta ∼\text{Normal}\left(0,\;\sigma_{\beta }^{2}\right)$$

with variance modeled using a common hyper‐prior:

$$\sigma_{\beta }^{2}∼\text{Gamma}\left(2,\;0.1\right)$$

(Chung, Rabe‐Hesketh, Dorie, Gelman, & Liu, 2013). The detection parameters were modeled using uninformed prior distributions:

$$\rho ,\phi ∼\text{Beta}\left(1,\;1\right).$$

Posterior distributions for all parameters were generated using the No‐U‐Turn Sampler (Homan & Gelman, 2014) in Stan software version 2.17 (Carpenter et al., 2017) using 8,000 Markov chain Monte Carlo (McMC) iterations, with the first 4,000 used as warm‐up. Annotated Stan code is available in Appendix S3. Convergence was evaluated by inspection of trace plots and r‐hat values (Gelman & Hill, 2006).

### 2.4 Ignoring detection error

To evaluate the effect of including detection error (*ρ* and *ϕ*) on our estimates of seroprevalence and demographic effects, we also ran models excluding these parameters. Specifically, for this set of models, we defined:

$$y_{\mathrm{it}}∼\text{Bernoulli}\left(\psi_{\mathrm{it}}\right).$$

The observed disease state, *y_it_*, was a function of the probability that an individual was seropositive for the pathogen (*ψ_it_*) and *ψ_it_* was a function of demographic information:

$$\text{logit}\left(\psi_{\mathrm{it}}\right)=x_{i}^{T}\beta .$$

### 2.5 Model comparison and validation

For each pathogen, we compared models using Watanabe–Akaike Information Criterion (WAIC) and selected the model with the lowest score (Hooten & Hobbs, 2015). We conducted posterior predictive checks by using the model to predict apparent seroprevalence ($\hat{\pi }$) and comparing it with the observed apparent seroprevalence (*π*_observed_); if we can predict the apparent seroprevalence from the model, then we assume that the predicted latent true seroprevalence is accurate. Specifically, we calculated Bayesian *p*‐values as the mean discrepancy between posterior predicted apparent seroprevalence and observed apparent seroprevalence (Gelman & Hill, 2006). We also conducted Pareto‐smoothed importance sampling leave‐one‐out cross‐validation (PSIS‐LOO; Vehtari, Gelman, & Gabry, 2017). We performed out‐of‐sample model validation to assess model performance by withholding 25% of the dataset as the test dataset and using the remaining samples (75%) as the training dataset (Gelman & Hill, 2006). Apparent seroprevalence was predicted in the test dataset for each pathogen in each year using the model trained on the training dataset. This was compared with apparent seroprevalence calculated for each pathogen in each year in the test dataset. The training and testing datasets were selected using conditional Latin hypercube sampling, conditioned on demographic parameters and year of sampling, to ensure that each dataset represented the demographic distribution of the entire dataset (Minasny & McBratney, 2006).

### 2.6 Literature search

We identified previous studies reporting pathogen seroprevalence in wild pigs in North America for the five pathogens using a systematic review of the peer‐reviewed literature. Our approach was based on the Preferred Reporting Items for Systematic Reviews and Meta‐Analyses (PRISMA) method (Liberati et al., 2009; Moher, Liberati, Tetzlaff, & Altman, 2009). To implement the search of the peer‐reviewed literature, we searched three databases (PubMed, Scopus, and Web of Science) for scientific publications reporting surveillance results, pathology, and case reports using a priori selected keywords described by Miller et al. (2017). We restricted our analyses to studies that were conducted on wild pigs from the United States and the five pathogens analyzed in this study. From each relevant paper, we recorded reported seroprevalence, sample size, and location of the study. If the paper evaluated demographic effects, we recorded the directional effect of sex and age class on seroprevalence. In order to compare differences in the reported seroprevalence from the literature with our predicted seroprevalence, we calculated 95% credible intervals (CrIs) using Jeffrey's priors in the Binom package (version 1.1‐1) in R (Dorai‐Raj, 2009).

## 3 RESULTS

Out‐of‐sample model validation (Gelman & Hill, 2006) revealed a good correlation between the apparent seroprevalence (i.e., the proportion of individuals that tested positive) in the test dataset and predicted apparent seroprevalence in the training dataset (Table 1; Appendix S4) except for IAVS, which had a correlation of −0.83. In 2010, the first year in which sera were tested for exposure to IAVS, apparent seroprevalence was much higher (15%) than in other years (6.9%) and only 2.6% of the samples were from this year. When we excluded samples from 2010 from the analysis of model validation, the correlation was 0.82. Similarly, Bayesian *p*‐values were close to .5 and median relative bias was close to 0 for all modeled pathogens, indicating good predictive capacity of the models (Table 1). PSIS‐LOO predicted that the shape parameter of the Pareto distribution ($\hat{k}$) was <0.5 for each pathogen indicating good predictive abilities of the model (Vehtari et al., 2017).

**Table 1:** Model validation results for models estimating the seroprevalence of each pathogen

Pathogen	Validation correlation	Bayesian *p*‐value	Median relative bias
Porcine Reproductive & Respiratory Syndrome (PRRS)	0.99	.47	−0.095
Pseudorabies Virus (PRV)	0.99	.55	−0.086
Influenza A Virus in Swine (IAVS)	−0.83 (0.82)^a^	.47	−0.056
Hepatitis E (HEV)	1	.44	−0.027
*Brucella* spp. (SB)	0.87	.49	0.092

a Excludes 2010, a year in which the model predicted apparent seroprevalence poorly. Apparent IAVS seroprevalence in 2010 was over 200% of that from the remaining years, and only 3% of samples for this pathogen were from 2010, so it was excluded from this measure of validation.

**Figure 1:**
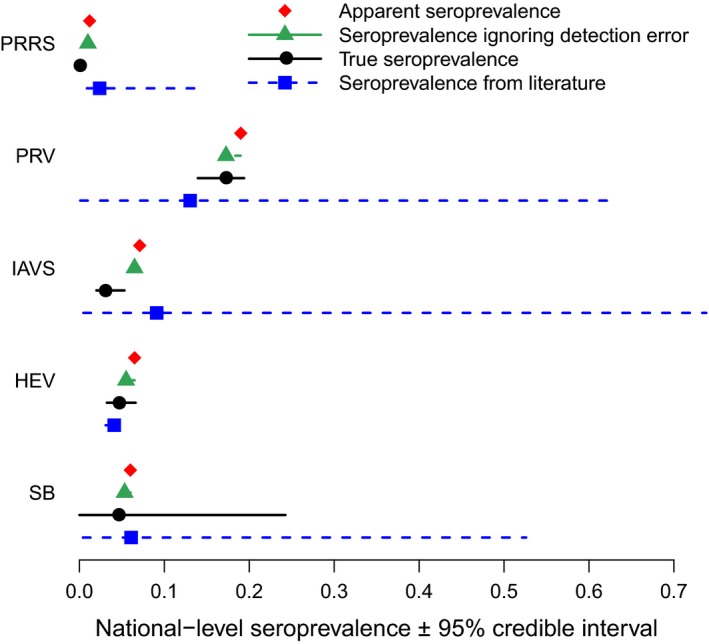
Comparison of seroprevalence predictions ignoring detection error with true seroprevalence predictions that account for detection error. The comparison includes porcine reproductive and respiratory syndrome virus (PRRS), pseudorabies virus (PRV), influenza A virus in swine (IAVS), hepatitis E virus (HEV), and *Brucella* spp. (SB). True seroprevalence that accounts for detection error was significantly different from seroprevalence predictions ignoring detection error for PRRS and IAVS. Medians are presented along with 95% credible intervals. Median seroprevalence from the literature was often close to apparent seroprevalence

**Figure 2:**
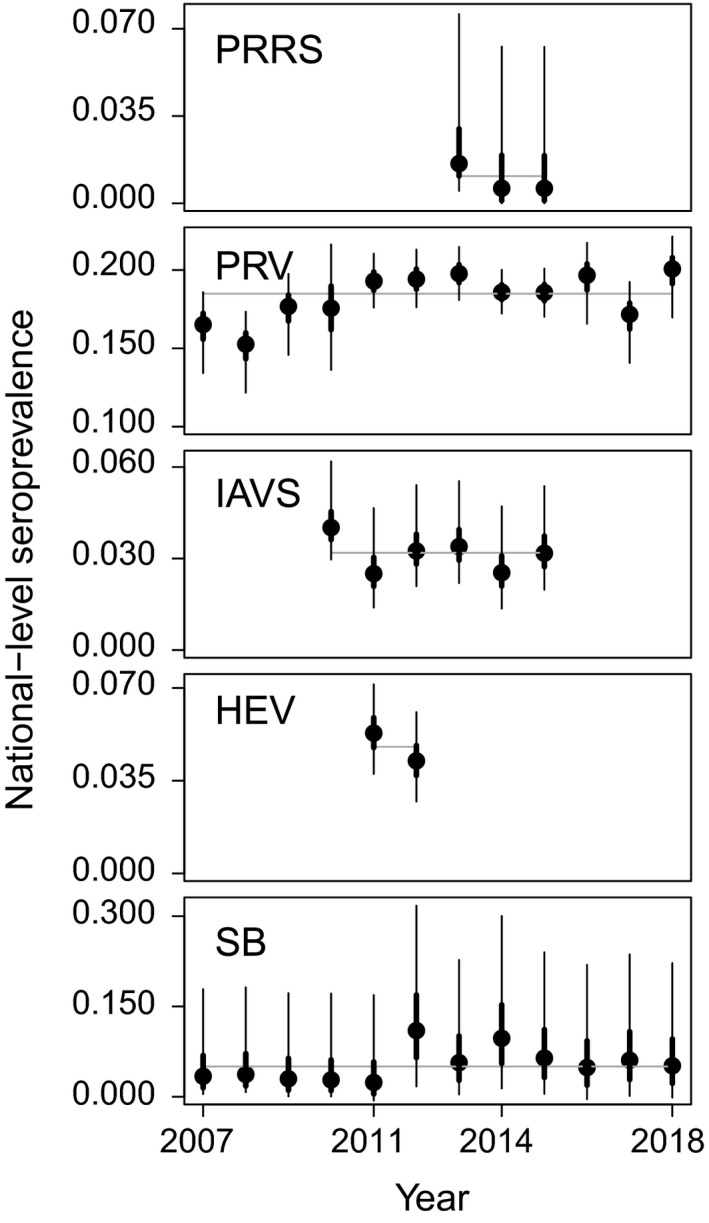
National‐level seroprevalence varied over time for porcine reproductive and respiratory syndrome virus (PRRS), pseudorabies virus (PRV), influenza A virus in swine (IAVS), hepatitis E virus (HEV), and *Brucella* spp. (SB). Points represent median of the posterior distribution, thicker bars represent the 50% credible intervals, and thin bars represent 95% credible intervals. The gray horizontal line represents the median seroprevalence across all years

Median predicted seroprevalence across all pathogens and years ranged from 0.006 to 0.17 and were significantly different from apparent seroprevalence—that is the credible intervals (CrIs) of predicted seroprevalence did not overlap with apparent seroprevalence—for PRRS and IAVS (Figure 1; Appendix S5). Median predicted annual seroprevalence varied through time for all pathogens ranging from 0.0006 for HEV to 0.20 for PRV (Figure 2; Appendix S6).

**Figure 3:**
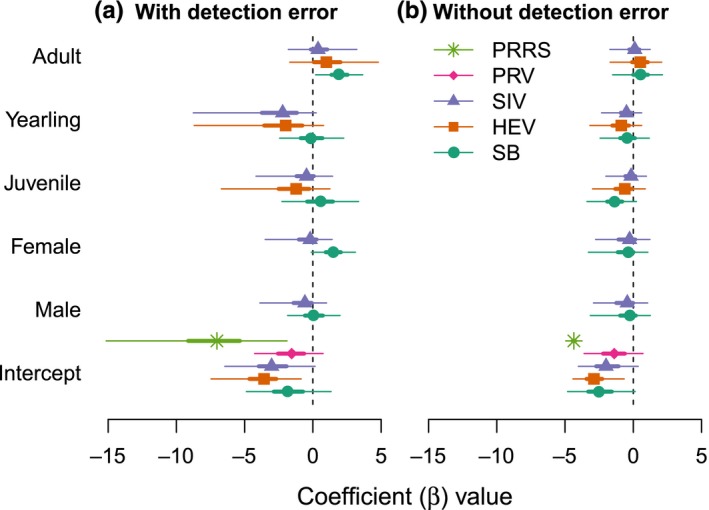
Posterior distributions for the effect of demographic parameters on pathogen seroprevalence for porcine reproductive and respiratory syndrome virus (PRRS), pseudorabies virus (PRV), influenza A virus in swine (IAVS), hepatitis E virus (HEV), and *Brucella* spp. (SB). Posteriors were generally wider when models incorporated detection error (a) than when models did not account for detection error (b). In some instances (e.g., the effect of juveniles on SB), the direction of the effect changed when detection error was ignored or the effect became insignificant (e.g., the effect of female and adult on SB). Points represent median of the posterior distribution, thicker bars represent the 50% credible intervals, and thin bars represent 95% credible intervals

**Figure 4:**
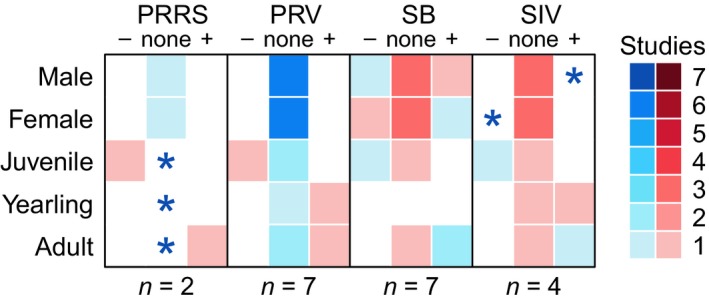
Agreement/disagreement between demographic effects found in this study with those of other studies for porcine reproductive and respiratory syndrome virus (PRRS), pseudorabies virus (PRV), influenza A virus in swine (IAVS), and *Brucella* spp. (SB). Blue boxes indicate that our study agreed with other studies from the literature, while red boxes represent disagreement. Asterisks are used if there were no studies in the literature finding the same directional effect as our study. The number of studies (*n*) reporting demographic effects for each pathogen is indicated below each panel. None of the studies represented here accounted for detection error in their estimates of seroprevalence. Only 20 of the 40 studies identified in our literature review attempted to evaluate demographic effects on prevalence

The best model for predicting pathogen seroprevalence, as determined by WAIC, was the intercept only model for two pathogens (PRRS and PRV). For IAVS and SB, the best model included age and sex, and for HEV, the best model included only an effect of age (Figure 3; Appendix S7). Most parameters had weak effects (the 95% CrI overlapped zero), despite being in the best model. However, there was a clear positive effect of the adult age class on seroprevalence of SB. The weak demographic effects we observed for most pathogens are similar to estimates from other researchers, who have generally found weak or no effects of demographic parameters on seroprevalence in wild pigs (Figure 4).

Sensitivity (*ρ*) and specificity (*ϕ*) are evaluated by the companies that manufacture the diagnostic tests. However, the sensitivity and specificity estimates are typically validated in domestic swine under controlled experimental conditions which may be different than those for wild swine. Our predictions of these parameters were similar to those previously reported for these diagnostic tests, with one exception: *ϕ* was higher than the estimate reported for IAVS from the manufacturer (Table 2; Appendix S2).

**Table 2:** Sensitivity (*ρ*) and specificity (*ϕ*) and 95% CrIs from models and from diagnostic tests used for each pathogen

Pathogen^a^	*ρ*	*ρ* diagnostic test	*ϕ*	*ϕ* diagnostic test
PRRS	0.92 (0.76, 0.99)	0.97 (0.91, 1.00)	0.99 (0.98, 0.99)	0.98 (0.90, 1.00)
PRV	0.98 (0.94, 1.00)	0.99 (0.96, 1.00)	0.98 (0.94, 1.00)	1.00 (1.00, 1.00)
IAVS	0.90 (0.84, 0.95)	0.90 (0.80, 0.98)	0.96 (0.95, 0.98)	0.85 (0.72, 0.94)
HEV	0.96 (0.86, 1.00)	1.00 (1.00, 1.00)	0.98 (0.96, 0.99)	0.98 (0.97, 0.99)
SB	0.93 (0.88, 0.97)	0.96 (0.92, 1.00)	0.96 (0.89, 0.99)	0.97 (0.92, 1.00)

a Pathogens evaluated were porcine reproductive and respiratory syndrome virus (PRRS), pseudorabies virus (PRV), influenza A virus in swine (IAVS), hepatitis E virus (HEV), and *Brucella* spp. (SB).

We found that models that did not include detection error (*ρ* and *ϕ*) predicted seroprevalence to be very similar to observed apparent seroprevalence (Figure 1). Model selection, as determined by WAIC, found the best demographic model for each pathogen was the same in both sets of models (Figure 3). However, the effects of demographic parameters in the models that did not account for detection error were weaker (CrIs were closer to zero), and for SB, *β* values for some parameters changed from positive to negative and from significant to insignificant (Figure 3).

Our literature search resulted in 40 relevant studies documenting seroprevalence for these five pathogens in wild pigs in the United States (Appendix S8). Reported seroprevalence estimates ranged from 0 to 0.75, with wide CrIs for all pathogens except HEV and IAVS (Figure 1). Nevertheless, median seroprevalence estimates from the literature were similar to median seroprevalence predictions from our study. Some studies reported demographic effects on pathogen seroprevalence (a total of 20 demographic analyses across all pathogens). Most (80%) of these analyses used *t* tests or similar analyses instead of estimating effect sizes using statistical models (i.e., GLMs or logistic regression; Appendix S8). Demographic effects in the literature differed by pathogen but were often weak or insignificant (Figure 4). Only one of the 40 studies (Pedersen, Miller, & Musante, 2018) used models incorporating sensitivity and specificity for the pathogen(s) analyzed.

## 4 DISCUSSION

Using hierarchical Bayesian models that account for uncertainty in detection, we estimated national‐level seroprevalence for five pathogens in wild pigs across time. Our modeling approach is useful for estimating temporal changes in pathogen prevalence and because our model structure accounted for sampling data that were nonsystematically collected using different diagnostic tests, our approach demonstrates the broad potential use of these types of data for estimating prevalence, changes in prevalence, risk factor associations, and detection error.

### 4.1 Pathogen seroprevalence in wild pigs

Our predictions of seroprevalence and associated risk factors have important implications for interpreting results of surveillance studies. We found that the national‐level seroprevalence of PRV in wild pigs was high and varied across years (median seroprevalence = 0.17 across all years). This is consistent with known epidemiology of the virus. Once wild pigs are infected with PRV, the virus establishes a lifelong latent infection accompanied by relatively decreased levels of neutralizing antibodies (Pedersen et al., 2013). The increasing seroprevalence in wild pig populations of North America indicates ongoing transmission that may present a risk to domestic animals and wildlife. Smith (2012) found that PRV was likely transmitted both sexually and nonsexually among wild pigs in North America. The potential role of nonsexual transmission may be a particularly important route of exposure and transmission of PRV to domestic animals and warrants additional investigation.

National‐level seroprevalence of SB varied considerably over time and generally had large 95% CrIs, with the upper bound ranging as high as 0.32. Fluctuating seroprevalence and large credible intervals indicate that there is likely temporal or regional variation in transmission that increases exposure of wild pigs to SB. Risks posed by SB‐infected wild pigs to humans and domestic animals likely vary greatly depending on location and time due to the different exposure rates and the distribution of feral swine. Accounting for this variation in the design of surveillance strategies may be particularly important.

Seroprevalences of PRRS, IAVS, and HEV were generally low across all years with some temporal variation (Figure 2), suggesting that these pathogens might be of less concern at a national scale, but variation observed over time may indicate some variation in regional transmission risk. This may be particularly relevant for PRRS, which had large credible intervals indicating that wild pig exposure to PRRS may vary regionally. However for all three of these pathogens, relatively little is known about the pathogen etiology and drivers of transmission and persistence for wild pigs of North America (Miller et al., 2017). Studies that elucidate these factors as well as risk factors associated with potential cross‐species transmission among wild pigs, humans, domestic animals, and wildlife are needed to better characterize potential risks posed by these three pathogens.

### 4.2 Demographic risk factors

The associations with demographic risk factors we found were similar to previous findings for PRV and PRRS. Our study supports previous studies that have found no significant effect of age class on seroprevalence of PRV (Hernández et al., 2018), although some studies have found higher exposure rates in older age classes in North America (Pedersen et al., 2013; Pirtle, Sacks, Nettles, & Rollor, 1989) and Europe (Lari et al., 2006). It is possible that since PRV often results in high mortality in piglets, they are not being sampled and thus artificially decreasing risk associations. However, none of the studies reporting associations with age accounted for detection errors, which may have biased their reported findings.

Contrary to the majority of previous studies (Figure 4), we found age and sex to be associated with increased risk of exposure for SB and IAVS. We found that adult males were more likely to be seropositive for IAVS (Figure 3). None of the previous four studies investigating differences in exposure risk for males and females have found an association, although these studies were conducted under different circumstances (Cleveland et al., 2017; Feng et al., 2014; Martin et al., 2017; Pedersen et al., 2017). While the effect of age on IAVS seroprevalence has been found to be unimportant in most studies (Figure 4, Appendix S8), our results supported two previous studies that found similar effects (Cleveland et al., 2017; Feng et al., 2014) and are consistent with IAVS risk factors in domestic pigs (Richt et al., 2003).

Similarly, we found associations with age and sex that differ from the majority of previous studies for SB (Figure 4). Higher seroprevalence of SB in females is consistent with one previous study (Musser, Schwartz, Srinath, & Waldrup, 2013) and could be attributed to the fact that SB is primarily a venereally transmitted disease that can lead to higher exposure and seroprevalence in females (Cross et al., 2009). Our findings indicate that exposure increases with age and is consistent with one previous study and more generally consistent with risk factors for other bacterial pathogens in wildlife (van der Leek et al., 1993; Pedersen et al., 2012).

### 4.3 Accounting for detection error in models of prevalence

Our results highlight the importance of incorporating detection error (i.e., sensitivity and specificity) into models of pathogen seroprevalence. Models that did not account for detection error resulted in predictions of seroprevalence that were significantly higher for both PRRS and IAVS (the 95% CrIs for models with and without detection error did not overlap; Figure 1). As expected, median seroprevalence modeled without detection error was nearly identical to apparent seroprevalence for all pathogens. Additionally, apparent seroprevalence was consistently higher than predicted true seroprevalence that accounted for detection error. This likely results from serological assays that are designed to maximize detection of a pathogen resulting in false‐positive animals. Serological assays, especially those used in domestic animals, are typically designed to be used in series with other diagnostic tests so false‐positive animals are subsequently identified with additional testing. For the five pathogens we investigated, using apparent prevalence would have resulted in a biased estimator.

Only one of the 40 (2.5%) studies analyzed used methods that incorporated detection error to predict prevalence (Pedersen et al., 2018). Using models to investigate associations between seroprevalence and risk factors that do not incorporate detection error effectively assumes that the pathogen is always detected when an individual is infected (*ρ* = 1) and that individuals testing positive are always infected (*ϕ* = 1). These assumptions are likely violated for most pathogens and diagnostic assays (Gilbert et al., 2013). We found that for all pathogens except PRV the lower 95% CrI for predicted sensitivity (*ρ*) was below 0.9 and predicted specificity (*ϕ*) generally had less variation but for all pathogens extended below 1 (Table 2, Appendix S9), indicating that this assumption (*ρ* = *ϕ* = 1) was violated. Therefore, we recommend future analyses of pathogen seroprevalence incorporate both sensitivity and specificity into models, especially if associations with risk factors are being investigated.

Including detection error may be particularly important for pathogens when poor diagnostic tools are available, diagnostic assays are designed with high sensitivity, or when results are obtained from different diagnostic laboratories that may be using different diagnostic assays. We found that when detection error was ignored the demographic effects were weaker, less significant, and occasionally in a different direction (Figure 3). While the effects of most demographic risk factors were insignificant, adults were significantly more likely to test positive for SB than other age classes in models accounting for detection error. However, when we ignored detection error in our models, this effect was no longer significant. Other demographic effects for SB (female, male, and juvenile), although statistically insignificant (the 95% CrIs overlapped 0), switched from positive to negative effects. Therefore, our results suggest that even in a study with weak demographic effects, ignoring detection error could change inference of which risk factors are associated with seroprevalence. This may be particularly important if these risk factors are being used to inform disease management or determine risk‐based targeted surveillance planning.

### 4.4 Limitations and extensions

Demographic effects on seroprevalence were weak or nonexistent for most pathogens, indicating that other risk factors might be important in predicting seroprevalence and in determining pathogen exposure. For PRRS and PRV, the best model included only the intercept, and for the other three pathogens, only one demographic effect was significantly different from zero (Figure 3). Additionally, the wide posterior distributions of the *β* values for most demographic parameters indicate that other variation was likely influencing exposure and seroprevalence. Previous studies have found similarly weak or nonexistent effects when analyzing how demographics contribute to seroprevalence (Figure 4; Appendix S8). While demographic risk factors may influence exposure risk at a local scale (Figure 4), spatial heterogeneity of these effects might cause them to diminish at the national scale. Previous studies have found that wild pig contact is strongly influenced by local scale factors of animal age, group membership, and distance between group home ranges (Pepin et al., 2016; Podgórski, Apollonio, & Keuling, 2018). Additionally, it is possible that strong spatial factors (e.g., climatic or ecological effects) might overshadow any demographic effects that would otherwise be observed.

Another important factor that might affect our predictions of seroprevalence and associations with demographic risk factors is the effect of demographic and environmental drivers on host detection probability. Probability of detecting a pathogen when present might depend on seasonality, climate, host age, etc., because these factors could interact with the effect of the pathogen on behavior or survival of infected individuals (Jennelle, Cooch, Conroy, & Senar, 2007). For example, deer infected with chronic wasting disease have been observed to be less active, less likely to migrate, and have home ranges that are 160% smaller than uninfected deer (Edmunds et al., 2018). This reduced activity can result in increased sampling of uninfected individuals, leading to lower estimates of prevalence (Nusser, Clark, Otis, & Huang, 2008). Conversely, infected animals may be more vulnerable to capture and sampled at a higher rate, which has been observed in some host–pathogen systems (Blanchong et al., 2012; Courchamp, Say, & Pontier, 2000). These effects of sampling bias resulting from infection status have been observed in many wildlife disease systems (Conner, McCarty, & Miller, 2000; Courchamp et al., 2000). However, the interaction with environmental conditions is rarely accounted for explicitly. Therefore, it would be valuable for future analyses to incorporate the effects of abiotic and biotic drivers on detection probabilities using methods such as those described by Jennelle et al. (2007).

## 5 CONCLUSIONS

It is becoming increasingly important in managing diseases that affect domestic animals and humans to understand the role of wildlife (Hassell et al., 2017). Seroprevalence is often used as a proxy for transmission risk and in many cases is the only measure of pathogen exposure available for wildlife (Pepin et al., 2017). While detection error is commonly addressed in ecological studies (Royle & Link, 2006) and there are many tools available to account for these errors (Jennelle et al., 2007; McClintock et al., 2010), it is still rarely accounted for in wildlife disease studies. We found that not accounting for detection error can significantly affect predictions of seroprevalence and risk factor associations. Future work to disentangle detection error, not only resulting from the diagnostic assay uncertainty but also resulting from host detection, is needed. Studies reporting associations with risk factors that do not account for detection error should be carefully interpreted.

## CONFLICT OF INTEREST

None declared.

## AUTHOR CONTRIBUTIONS

MAT and RSM developed the model, interpreted the results, and led the writing; KP collected samples and assisted in writing and interpretation of results; MAT programmed the model and conducted analyses; all authors contributed critically to drafts and gave final approval for publication.

## Data Availability

Pathogen sampling data are available in the Dryad Digital Repository (https://doi.org/10.5061/dryad.8p25386; Tabak, Pedersen, & Miller, 2019).
