# Supplementary figures and images for: Detection error influences both temporal seroprevalence predictions and risk factors associations in wildlife disease models
Source: Ecol Evol. 2019 Aug 27;9(18):10404–14. doi: 10.1002/ece3.5558 (PMC6787870; doi:10.1002/ece3.5558)

a) Distribution of samples by state

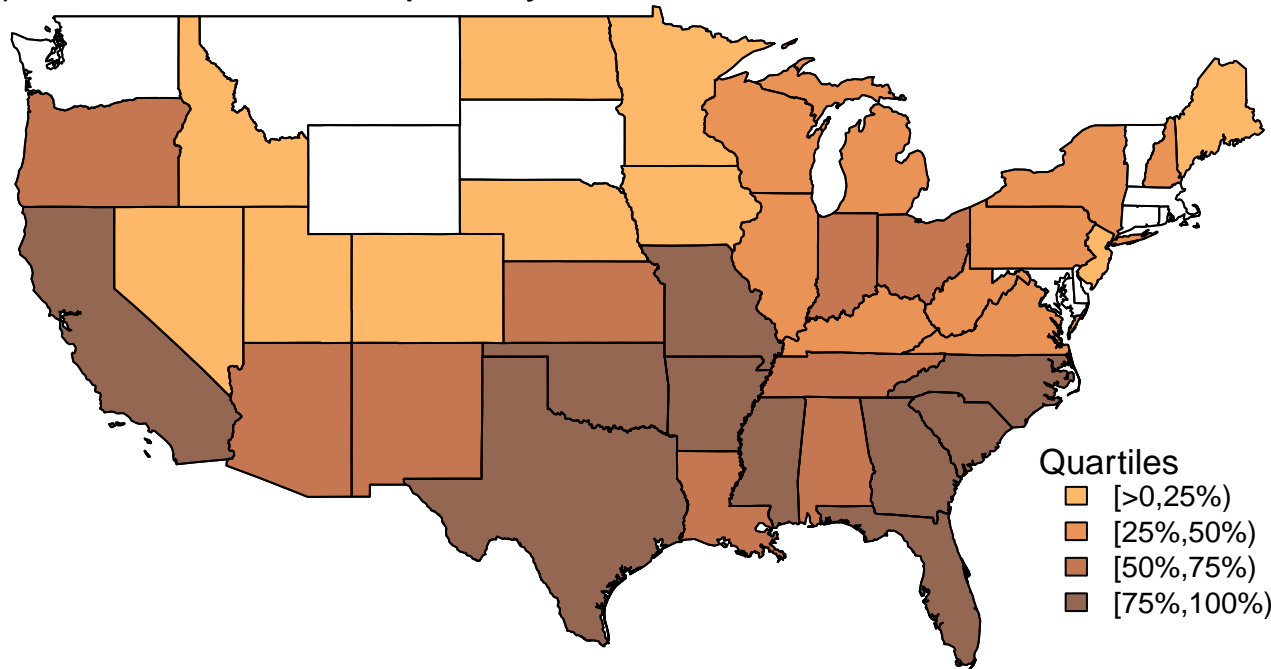

b) Total number of samples per year

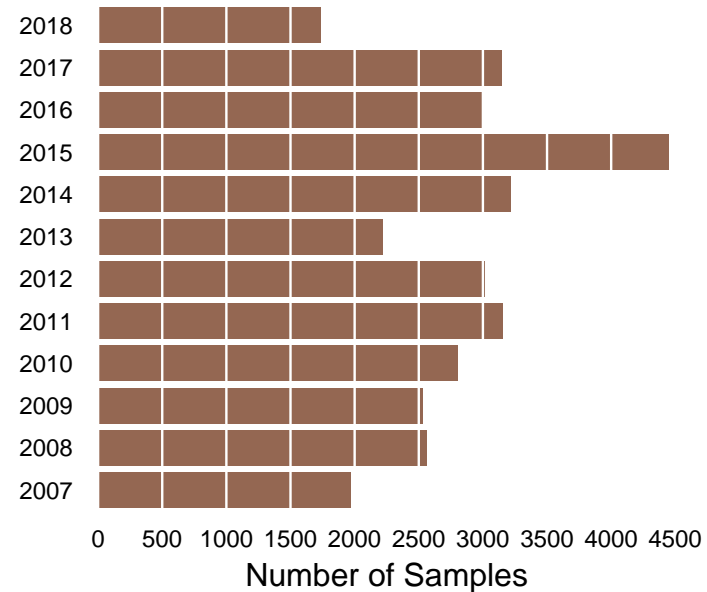

Supplement: Supplementary file 1 [file ECE3-9-10404-s001.pdf]

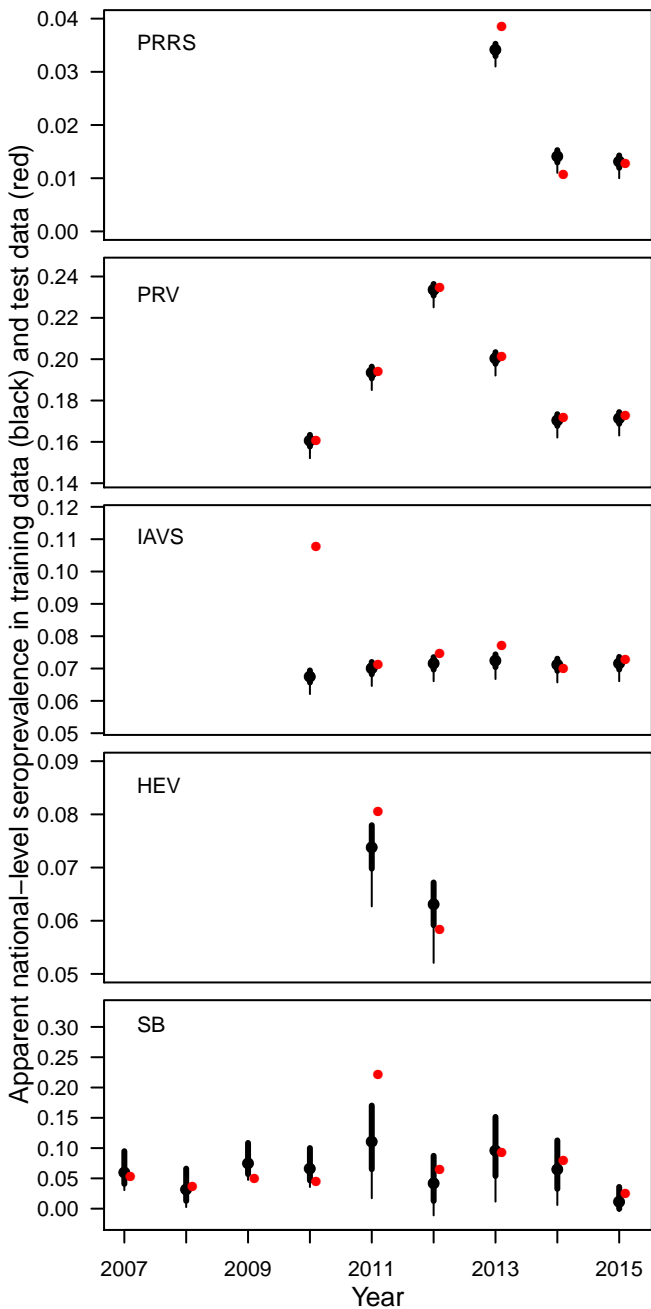

Supplement: Supplementary file 4 [file ECE3-9-10404-s004.pdf]

$\rho$  $\phi$ 

PRRS

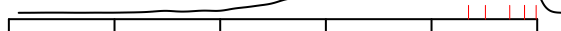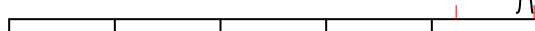

PRV

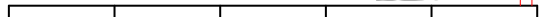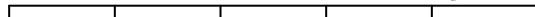

IAVS

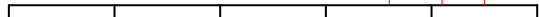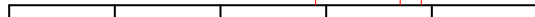

HEV

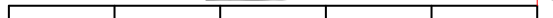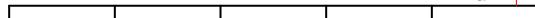

SB

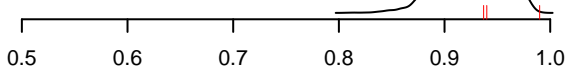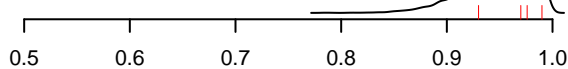

Supplement: Supplementary file 9 [file ECE3-9-10404-s009.pdf]
